# Visualization of flow in the ascending aorta: bicuspid aortic valves compared to tricuspid aortic valves

**DOI:** 10.1186/1532-429X-13-S1-P384

**Published:** 2011-02-02

**Authors:** Christian Meierhofer, Christine Lyko, Eike Philipp Schneider, Andrea Hutter, Heiko Stern, Stefan Martinoff, Alfred Hager, John Hess, Michael Markl, Sohrab Fratz

**Affiliations:** 1 German Heart Center Munich, Munich, Germany; 2 University Hospital Freiburg, Freiburg, Germany

## Study’s objective

The aim of this prospective matched-pair study was to compare different 3D flow patterns in the ascending aorta caused by native bicuspid aortic valves (BAV) compared to tricuspid aortic valves (TAV).

## Background

The presence of bicuspid aortic valves may account for dilation of the ascending aorta and increase morbidity and mortality in this patient group. There is an ongoing debate on the cause of increasing diameters of the ascending aorta. Intrinsic wall abnormalities due to genetic development have been discussed as well as altered hemodynamic properties due to different valve patterns.

## Methods

Our prospective matched-pair study included 18 patients (median age 25 years, range 8 - 44 years) with a native bicuspid aortic valve and normal diameters of the ascending aorta, without stenosis or insufficiency of the aortic valve and no coarctation of the aorta. All of the 18 patients were age- and sex- matched with 18 controls with a tricuspid aortic valve. All patients with BAV were otherwise healthy without any cardiovascular disease.

Both groups underwent time-resolved flow-sensitive 4D CMR for the individual evaluation of 3D flow pattern of the ascending aorta (spatial resolution = 2.1 x 1.7 x 2.5 mm^3^, temporal resolution = 39.2 ms). All data sets were evaluated independently by three blinded investigators. According to a predefined classification, the helical flow patterns were graded.

## Results

**Figure 1:**
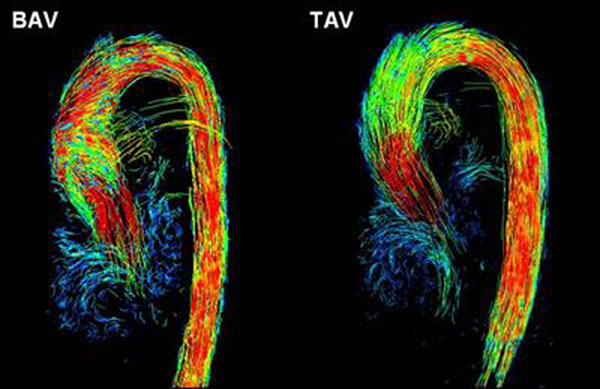
Matched pair (left BAV, right TAV) of 32-year old women.

The blinded evaluation resulted in 90% in correct classification of BAV or TAV. In the BAV group, 85 % showed a flow pattern with a high-grade helix formation in the ascending aorta. The matched pairs showed a statistically significant difference of the flow patterns. Figure 1.

## Conclusions

In about 90 % the aortic valve morphology BAV or TAV can be accurately predicted by the individual flow pattern measured by 4D CMR. Without concomitant lesions and normal aortic dimensions most of the patients with BAV show a pathologic flow pattern. We suggest that this method may help to distinguish patients with BAV at risk for aortic dilatation.

